# Immune Modulation for Enzyme Replacement Therapy in A Female Patient With Hunter Syndrome

**DOI:** 10.3389/fimmu.2020.01000

**Published:** 2020-05-21

**Authors:** Daniel C. Julien, Kara Woolgar, Laura Pollard, Holly Miller, Ankit Desai, Kristin Lindstrom, Priya S. Kishnani

**Affiliations:** ^1^Center for Cancer and Blood Disorders, Phoenix Children's Hospital, Phoenix, AZ, United States; ^2^Division of Genetics and Metabolism, Phoenix Children's Hospital, Phoenix, AZ, United States; ^3^Division of Medical Genetics, Greenwood, SC, United States; ^4^Division of Medical Genetics, Department of Pediatrics, Duke University Medical Center, Durham, NC, United States

**Keywords:** MPS II, hunter syndrome, immune modulation, enzyme replacement therapy, neutralizing antibodies

## Abstract

A 3.5 year old Hispanic female presented with signs and symptoms concerning for MPS II (Hunter Syndrome). The diagnosis of MPS II was confirmed by enzyme and molecular testing. Genetic evaluation revealed undetectable plasma iduronate-2-sulfatase enzyme activity and an inversion between intron 7 of the *IDS* gene and a region near exon 3 of *IDS-2*. This inversion is the molecular cause for ~8% of cases of MPS II and often results in a severe phenotype. X-inactivation studies revealed an inactivation ratio of 100:0. Given the patient's undetectable enzyme level, in combination with a severe IDS gene mutation, classic features at time of presentation, and the significantly skewed X inactivation, there was concern that she was at high risk of developing high and sustained antibody titers to idursulfase which would limit her benefit from enzyme replacement therapy (ERT). Anti-drug neutralizing antibodies to idursulfase have been associated with reduced systemic exposure to idursulfase and poorer clinical outcomes. Therefore, the decision was made to concurrently treat the patient with immune tolerance induction therapy during the first month of treatment with idursulfase in order to decrease the risk of developing high sustained antibody titers. The immune tolerance induction protocol consisted of rituximab weekly for 4 weeks, methotrexate three times a week for 3 weeks and monthly IVIG through B-cell and immunoglobulin recovery. Immune tolerance induction was initiated concurrently with the start of ERT. The patient had no significant adverse effects related to undergoing immune tolerance induction therapy and two and half years later is doing well with significantly reduced urine glycosaminoglycans and very low anti-drug antibody titers. This immune tolerance induction protocol could be considered for other patients with MPS II as well as patients with other lysosomal storage disorders who are starting on enzyme replacement therapy and are at high risk of developing neutralizing anti-drug antibodies.

## Introduction

Mucopolysaccharidosis type II (MPS II; OMIM # 309900), also known as Hunter syndrome, is a rare, X-linked lysosomal storage disease caused by a deficiency in the enzyme iduronate-2-sulfatase, encoded by the IDS gene (1–3). Deficiency of iduronate-2-sulfatase leads to accumulation of glycosaminoglycans dermatan and heparan sulfate in lysosomes, resulting in cellular and tissue damage, enlargement of the affected organs and ultimately disruption of normal cellular physiology and organ dysfunction (2). As MPS II is an X-linked disorder, it predominately affects males with an estimated occurrence of 1 in 130,000–170,000 live male births in those of Caucasian descent (4). Females may also rarely be affected due to certain X-chromosome deletions, chromosomal translocations, or unequal X-chromosome inactivation (5, 6). The clinical manifestations and phenotype of MPS II vary widely. Factors such as the age of presentation, severity of the disease, and rate of disease progression are correlated with the severity of a specific mutation. Indeed, over 450 unique *IDS* mutations have been documented in the literature (7). One of the most significant distinctions in the severity of MPS II is the presence or absence of cognitive impairment (8). Patients with the more severe form of the disease have a cognitive decline in the first few years of life, and often do not survive past 15 years of age, while those with the attenuated form of MPS II may have normal intelligence and live well into adulthood (8). Typically patients with absent or very little enzyme activity such as those with large gene deletions, gene rearrangements as well as nonsense, frameshift, and splice-site variants have a more severe phenotype compared to those with less significant mutations such as point mutations or deletions (9). The common clinical features of MPS II also include: joint stiffness and joint contractures leading to decreased range of motion, coarsening of the facies, macrocephaly, hepatomegaly, cardiomegaly with heart valve dysfunction, decreased growth velocity, reduced endurance, and decreased pulmonary function (10).

Historically, the management of MPS II had focused on relieving the symptoms of the disorder through surgical interventions and other supportive care measures (11). In the 1980's hematopoietic stem cell transplantation (HSCT), which had been successfully used in the treatment of mucopolysacharodosis type I, was first utilized for the treatment of MPS II, though with varying success (12–16). One limitation of HSCT in patients with MPS II is transplant-related morbidity or mortality. A review of SER data from patients that had undergone HSCT for MPS II between 1982 and 2007 revealed a 78% overall survival and 62% event free survival, but this data does not take into account the patients' age, phenotype, donor status or transplant protocols (17–19). However, newer data such as that from Japan, where HSCT for MPS II is routinely offered, shows a 5 year survival rate of 88.5% (20). Also, 11 of the 17 patients with MPS II that received HSCT in this study had stabilization of brain atrophy and were less likely to have speech deterioration compared to those who were untreated (20). However, HSCT prep regimens require the use of strong chemotherapeutics as well as radiation in some protocols. Long term complications from HSCT include graft vs. host disease, increased risk of malignancy, cataracts, as well as decreased fertility, to name a few.

In 2006 the treatment of MPS II was revolutionized with the Food and Drug Administration's approval of the intravenous (IV) infusion of idursulfase (Elaprase®, Shire HGT, Lexington, MA) for enzyme replacement therapy (ERT) in confirmed cases of MPS II. Though exogenous ERT is not able to efficiently cross the blood–brain barrier and therefore is not able to prevent the cognitive decline associated with the disease, clinical trials of intrathecal administration of idursulfase are underway (21, 22). ERT has been shown to be beneficial for various other aspects of the disease. For example patients receiving ERT have an improvement in their endurance on the 6-min walk test, reduction in liver and spleen size, improved pulmonary functional status, and reduction in urinary GAGs (23, 24). With the proven success of ERT, it has become the standard therapy for MPS II.

Though ERT has changed the landscape of management of MPS II, there are some significant challenges that can limit its clinical efficacy. ERT requires chronic (usually 0.5 mg/kg weekly) infusions of idursulfase, which may lead to the development of anti-drug antibodies to the exogenous enzyme. Although the presence of anti-idursulfase antibodies does not always translate into a confirmed decrease in the efficacy of ERT, 50% of treated patients go on to develop IgG antibodies within the first year of treatment (23–25). Of patients developing antibodies, 21% to 35% also have or go on to develop neutralizing IgG anti-drug antibodies to idursulfase (26, 27). Neutralizing anti-drug antibodies have been associated with reduced systemic exposure to idursulfase and subsequently less of a reduction of urinary GAGs, decreased improvements in pulmonary function, and diminished reduction in liver volume (7, 25, 27–29). With limited alternative therapeutic options, the development of strategies to eradicate or prevent the formation of neutralizing anti-drug antibodies is of vital importance. Though there is limited data in patients with MPS II, immune tolerance induction protocols using a combination of cytotoxic and immune suppressive agents have been successfully utilized in other types of lysosomal storage disorders (LSDs), particularly Pompe disease (30, 31). In this case study, we describe to our knowledge the first female patient with MPS II with zero *IDS* gene activity due to skewed X-inactivation to safely undergo concurrent immune tolerance induction therapy at the time of initiation of ERT with idursulfase.

## Case Report

A 3.5 year old ex 36 week female born via C-section after premature rupture of membranes presented to our hospital's Department of Genetics and Metabolism for evaluation of developmental delay. She was born to a 32 year old G3P3 mother and 32 year old father. Parents are non-consanguineous and both of Mexican descent. The patient was born via repeat cesarean section and spent ~2 weeks in the NICU for respiratory distress. Her past medical history was significant for global delay of milestones, snoring, and at time of presentation had recently been diagnosed with autism and attachment disorder. She had not had any prior surgical procedures. At the time of presentation, the patient was 3.5 years old and noted to have global developmental delay, coarse facies, macrocephaly, macroglossia, symmetric joint contractures, and hepatomegaly. Her head circumference was 53.8 cm (>99%), height was 108.8 cm (>99%), and weight was 26.9 kg (>99%). Brain MRI revealed delayed myelination, mildly low parenchymal volume, and mild brachycephaly. She was also noted to have palpable hepatosplenomegaly on exam. A comprehensive 4-generation family medical history revealed no other similarly affected individuals. Fragile X testing had been done and was normal. Based on the patient's features, exam, and imaging it was suspected that she may have a mucopolysaccharodosis disorder. Work up revealed elevated urine total glycosaminoglycans at 72.56 mg/mmol creatinine (4.5x ULN) with heparan sulfate 70.3 g/mol creatinine (12x ULN) and dermatan sulfate 58.07 g/mol creatinine (7x ULN). Functional enzyme testing results showed undetectable plasma iduronate-2-sulfatase enzyme activity and gene sequencing of the iduronate-2-sulfatase gene found an inversion between intron 7 and a region near exon 3, consistent with Hunter syndrome (MPS II). Of note, this specific mutation is responsible for ~8% of cases of MPS II. Interestingly, she was found to have a normal karyotype and chromosome microarray but was found to have X-chromosome inactivation at a ratio of 100:0. Echocardiogram revealed structurally normal cardiac anatomy as well as normal right and left ventricular size, but mildly thickened mitral and aortic valves. Sleep study revealed delayed sleep latency but no evidence of obstructive sleep apnea.

Due to the patient's *IDS* gene mutation, significantly skewed X-inactivation, as well as the severity of her phenotype with developmental delay, she was felt to likely have the severe/classic form of MPS II. Also, due to her age at the time of diagnosis, it was felt that she was not a good candidate for HSCT. Furthermore, there was a concern that she was at high risk of developing high and sustained anti-drug antibody titers upon initiation of idursulfase ERT, which would in turn limit the action or response to ERT. If the patient did go on to develop neutralizing anti-drug antibodies, there are limited alternative therapeutic options including eligibility for clinical trials, which would likely be further limited due to the patient being female.

With these factors in mind, the decision was made to start weekly idursulfase (0.5 mg/kg) with concurrent immune tolerance induction during the first month of ERT therapy with the goal of reducing the likelihood that the patient would go on to develop high sustained neutralizing antibody titers. The immune tolerance induction protocol utilized was first described by Kishnani et al. in cross-reactive immunologic material (CRIM)-negative infantile Pompe disease patients and as outlined in (Table 1) consisted of rituximab 375 mg/m^2^/dose IV weekly for 4 weeks (4 total doses), methotrexate 0.4 mg/kg orally three times weekly for 3 weeks (9 total doses), and IVIG 500 mg/kg IV weekly for 4 doses and then monthly through B-cell recovery (32, 33). The patient did not experience any infusion-related reactions with IV rituximab. Labs throughout treatment were within normal limits including her absolute neutrophil count, which ranged between 1,720 and 2,900 (NL > 1,500). Her immunoglobin levels, as well as a lymphocyte subset panel, were monitored monthly to determine B-cell recovery. While receiving methotrexate she had a very minimal elevation of liver function enzymes. Her maximum AST was 49 (1.3x ULN) and her maximum ALT was 115 (1.8x ULN). Total bilirubin was normal at all time points. The patient experienced no instances of illness necessitating emergency room evaluation or hospitalization while undergoing immune tolerance induction.

**Table 1:** Immune tolerance induction treatment protocol. ITI was administered shortly prior to Eleprase infusion.

**Week 1**	**Week 2**	**Week 3**	**Week 4**
Day 1:Rituximab IV	Day 1:Rituximab IV	Day 1:Rituximab IV	Day 1:Rituximab IV
Day 2:MTX SQ, Elaprase IV, IVIG IV	Day 2: MTX SQ, Elaprase IV, IVIG IV	Day 2: MTX SQ, Elaprase IV, IVIG IV	Day 2: Eleprase IV, IVIG IV
Day 3: MTX oral	Day 3: MTX oral	Day 3: MTX oral	
Day 4: MTX oral	Day 4: MTX oral	Day 4: MTX oral	

*Rituximab was administred at 375 mg/m^2^/dose IV weekly for 4 weeks (4 total doses), methotrexate 0.4 mg/kg orally three times weekly for 3 weeks (9 total doses), and IVIG was given 500 mg/kg IV once weekly for 4 doses and then monthly for 6 months until B-cell/plasma cell recovery. After week 4, Elaprase was continued weekly*.

Nearly 2.5 years after completion of the immune tolerance induction protocol she has been clinically stable. She has continued on weekly ERT and has had some notable improvement in her clinical status, including improved range of motion in her joints, a decrease in snoring and noisy breathing, and no evidence of worsening cardiomegaly, though has persistent valvular disease. MRI at time of initial presentation showed delayed myelination for age and global mildly low parenchymal volume, but no evidence of hydrocephalus and she has not had any seizures. In terms of cognitive ability, the patient remains substantially delayed (she is not toilet trained and is able to only count to the number 2), but she has made some improvements in language skills and is now able to form simple sentences, while before she communicated with single words or pointing. At this time she has an IgG titer of 1:160, these are very low titers, and which have no negative impact on clinical status.

**Figure 1:**
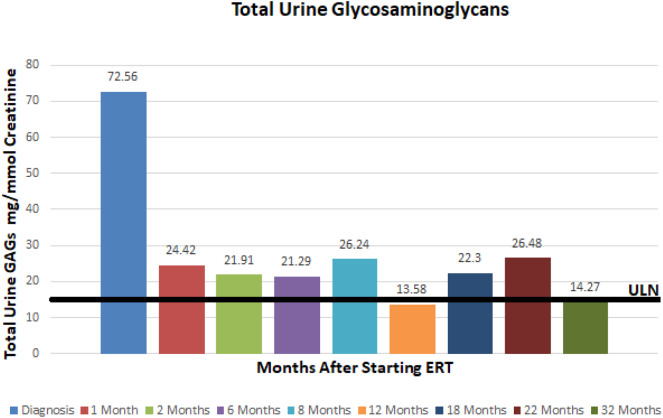
Total urine glycosaminoglycans at baseline and after starting enzyme replacement therapy. Normal range 0–16 mg/mmol creatinine.

**Figure 2:**
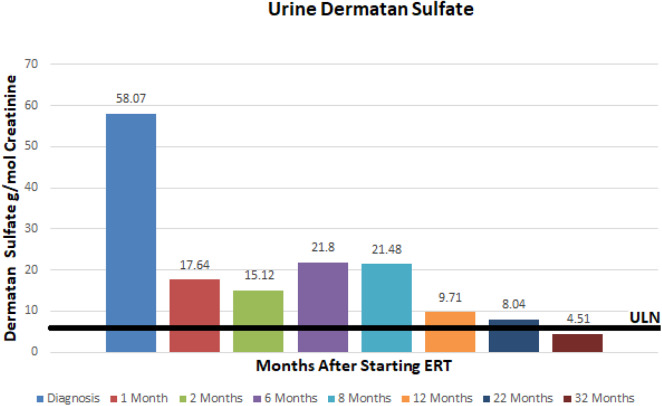
Urine dermatan sulfate at baseline and after initiation of enzyme replacement therapy. Normal range 0–7.93 g/mmol creatinine.

**Figure 3:**
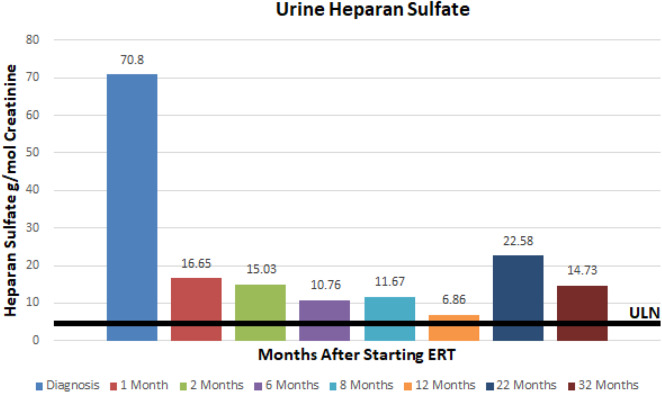
Urine heparan sulfate at baseline and after initiation of enzyme replacement therapy. Normal range 0–5.71 g/mmol creatinine.

At ~3–6 month intervals she has undergone monitoring of urine glycosaminoglycans, serum IgE, serum IgG, and anti-drug antibodies. Urine total glycosaminoglycans, dermatan sulfate, and heparan sulfate have significantly improved while receiving ERT as illustrated in (Figures 1–3), respectively. Serum IgE levels have been within normal limits at each time point monitored and clinically she has not had any signs or symptoms of allergic reaction or anaphylaxis during subsequent idursulfase infusions. Serum IgG levels have been within normal limits at each time point. The patient's anti-drug antibodies had been negative, however, her two most recent anti-drug antibody titers conducted by Convance Central Lab Services showed mild seroconversion (1:40 and 1:160, respectively) and have not had any negative impact on clinical status. This is in stark contrast to the high antibody titers in patients with MPS II, which typically range from 1:1000 to 1:100,000.

## Discussion

MPS II is a rare, X-linked metabolic disorder caused by a deficiency of the lysosomal enzyme iduronate-2-sulfatase. Though it predominately affects males, there are rare cases in females such as the patient described in this case. The mainstay of treatment is ERT with idursulfase, which has been shown to improve lung function, liver and spleen size, increase 6 min walk time distance, and improve shoulder range of motion, ultimately resulting in an improved quality of life. However, the clinical trial for approval of idursulfase as well as several other studies have shown that patients with large gene deletions, gene rearrangements (such as our patient), and nonsense, frameshift, and splice site variants are at an increased risk of developing neutralizing anti-idursulfase antibodies compared to those with smaller gene alterations like missense mutations (21, 27, 34). Neutralizing anti-drug antibodies are associated with poor clinical outcomes such as decreased pulmonary function due to antibody-mediated inhibition of idursulfase therapeutic activity as well-decreased pulmonary expansion from hepatosplenomegaly and elevated urine glycosaminoglycans (27, 29, 34).

**Figure 4:**
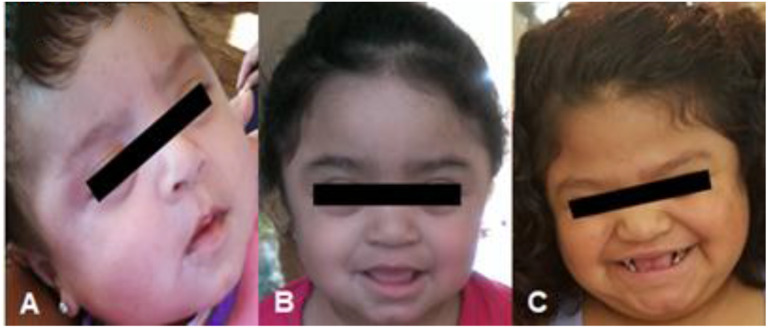
Patient as an **(A)** infant, **(B)** toddler, and **(C)** at time of presentation. Consent for use of patient photos was provided by the patient's parent/legal guardian.

In this case presentation, we report on our experience with a short course of immune tolerance induction therapy in a female patient with severe MPS II as an approach to prevent the development of high sustained neutralizing antibodies. Due to the patient's *IDS* gene mutation, significantly skewed X-inactivation, as well as the severity of her phenotype with developmental delay, she was felt to likely have the severe/classic form of MPS II, as is illustrated in the photos provided by the patient's legal guardian in (Figure 4). Typically patients with absent or very little enzyme activity such as those with large gene deletions, gene rearrangements as well as nonsense, frameshift, and splice-site variants have a more severe phenotype compared to those with less significant mutations such as point mutations or deletions (9). Furthermore, those with the severe type of MPS II are more likely to develop anti-drug antibodies due to the lack of exposure to native enzyme. This placed her at high risk of developing high and sustained anti-drug antibody titers upon initiation of idursulfase ERT, which would in turn limit the action or response to ERT. If the patient did go on to develop neutralizing anti-drug antibodies, there are limited alternative therapeutic options including eligibility for clinical trials, which may have been limited due to patient's gender. HSCT was ruled out due to her age at the time of diagnosis and severity of symptoms.

Previous studies in patients with infantile Pompe disease who receive immune tolerance regimens at the initiation of ERT are less likely to develop neutralizing anti-drug antibodies (31). Unfortunately, once neutralizing anti-drug antibodies have developed, it is often extremely challenging to completely eliminate them so the focus has been on prevention of the development of antibodies. Utilizing a similar combination of immune suppressant medications that has been safe and used successfully for prevention of development of antibody titers in CRIM-negative infantile Pompe patients, the immune tolerance induction protocol used for this patient consisted of weekly infusion of the anti-CD20 monoclonal antibody rituximab, methotrexate, and IVIG. Some clinicians have used a different anti-CD20 monoclonal antibody called ofatumumab (29), which is a completely humanized antibody, in patients with MPS II that have already developed neutralizing anti-drug antibodies. Bortezomib, a proteasome inhibitor, has also been used due to its ability to eliminate long-lasting plasma cells. Prior studies of bortezomib have been conducted in patients with infantile Pompe disease patients who had high sustained antibody titers and a decline in clinical response to ERT. Bortezomib was felt to be safe and well-tolerated in these patients (35, 36). Additionally, corticosteroids such as dexamethasone have been used as part of the backbone of some immune tolerance induction protocols. The effects of corticosteroids on the immune system are many, but in general they are immune suppressive and have a synergistic effect in combination with medications like bortezomib in the rapid reduction of plasma cell populations (35).

Burton et al. have previously reported the effect of an immune tolerance induction protocol consisting of ofatumumab, methotrexate, intermittent corticosteroids, and IVIG in a patient with MPS II who already had high levels of neutralizing anti-drug antibodies to idursulfase (29). Though the patient did have a significant decline in neutralizing anti-drug antibodies over a 1.5 year period, full eradication was not achieved and urinary GAGs were only modestly reduced. As the patient continued to have decreased, but persistent neutralizing anti-drug antibodies, he continued on weekly IV methotrexate, IVIG, and IV rituximab every 3 months for a total of 1 year. Fortunately, the patient in this study had not been previously treated with ERT and she was able to undergo immune tolerance induction therapy concurrently with the induction of ERT. She experienced no obvious side effects from immune tolerance induction therapy including no illnesses or hospitalizations while receiving immune tolerance induction therapy. She was however noted to have a transient small rise in liver function enzymes, which may be related to the low doses of methotrexate she received, though could be from a variety of other causes such as a viral illness at the time of her lab draws as well.

This patient, as well as other patients with LSDs that have undergone immune tolerance induction therapy in the naïve setting, demonstrate the efficacy of immune tolerance induction strategies in preventing neutralizing anti-drug antibodies. The scientific community has made strides in recognizing the genetic alterations that put patients with LSDs and other metabolic disorders that are managed with ERT at risk of developing neutralizing anti-drug antibodies. However, several challenges and questions remain. For example, the testing for anti-drug antibodies is sometimes difficult to obtain and may have a slow turnaround time. The ideal timeframe of when to carryout immune tolerance induction therapy has not been identified, though it serves to consider that at initiation ERT could have the greatest chance of preventing the formation of neutralizing anti-drug antibodies, as this is when the patient's immune system is initially challenged with medication and before the development of memory B cells and long-lived plasma cells. Several combinations of immunosuppressive agents have been utilized in immune tolerance induction protocols, but it is unclear if one specific combination is superior. Also, though we understand that large genetic alterations lead to the development of neutralizing anti-drug antibodies, the immunologic factors that contribute to antibody development are complex and the precise interplay of factors remains unclear. As we gain further knowledge and experience in the use of immune tolerance induction therapy in LSDs these questions will be answered and will hopefully ultimately result in improved patient outcomes.

## Data Availability Statement

All datasets generated for this study are included in the article/supplementary material.

## Ethics Statement

Written informed consent was obtained from the minor(s)' legal guardian/next of kin for the publication of any potentially identifiable images or data included in this article.

## Author Contributions

DJ was the primary author and wrote the manuscript and prepared tables and figures. KW, LP, HM, AD, and KL were secondary authors. PK was the final/supervising author.

## Conflict of Interest

The authors declare that the research was conducted in the absence of any commercial or financial relationships that could be construed as a potential conflict of interest.
